# Analysis of combined screening results of the hearing and deafness genes in 10,754 newborns

**DOI:** 10.1515/med-2025-1329

**Published:** 2026-01-28

**Authors:** Jiejing Lian, Ting Wu, Aiyu Jin, Hao Wang, Zhaojun Cheng

**Affiliations:** Prenatal Diagnosis Center, Hangzhou Women’s Hospital, Hanghzou, China; Child Health Department, Hangzhou Women’s Hospital, Hanghzou, China; Reproductive Medicine Center, Hangzhou Women’s Hospital, Hanghzou, China

**Keywords:** hearing, newborns, non-syndromic deafness, hearing screening, genetic testing for deafness

## Abstract

**Objectives:**

This study aimed to evaluate the significance of integrating hearing screening with genetic testing for hereditary deafness.

**Methods:**

A retrospective analysis was performed on the hearing screening and genetic testing outcomes of 10,754 newborns delivered at Hangzhou Women’s Hospital from June 2020 to December 2022. Hearing evaluations were conducted using transiently evoked otoacoustic emissions (TEOAE) and automated auditory brainstem response (AABR). For genetic testing, dried blood spots were collected, and 15 variants across four genes (GJB2, GJB3, SLC26A4, and mtDNA 12S rRNA) were examined using a DNA microarray platform.

**Results:**

Among the 10,754 infants, the most commonly detected mutations were GJB2c.235delC (47.26 %) and SLC26A4 IVS7-2 A>G (21.17 %). A total of 62 infants (0.58 %) were referred for additional hearing assessments, while 529 (4.92 %) tested positive for genetic mutations (including heterozygous, homozygous, or compound heterozygous variants, as well as mtDNA 12S rRNA homoplasmy), with 522 (4.85 %) passing the hearing screening. Three infants (0.028 %) had two variants in GJB2 (either homozygous or compound heterozygous), and one of these infants passed the newborn hearing test. Additionally, 33 infants (0.307 %) had the MT-RNR1 variant (m.1555A>G or m.1494C>T), all of whom passed the hearing screening.

**Conclusions:**

The most prevalent mutations associated with deafness were identified as GJB2 c.235delC, SLC26A4 IVS7-2 A>G, and m.1555A>G. The combination of hearing screening and genetic testing for deafness effectively identifies high-risk children with hereditary deafness for further intervention.

## Introduction

Deafness is primarily attributed to factors such as low immunity, infections, trauma, improper medication use, malformations, genetic influences, and other physiological issues, all of which significantly impact the health and quality of life of affected individuals. The World Health Organization reports a global incidence of 3–10 % for neonatal hearing loss [1], [2], [3]. Recent studies highlight that 60 % of congenital deafness cases are linked to genetic factors, with most hereditary deafness cases being non-syndromic, typically presenting as sensorineural deafness [4]. Traditional neonatal audiological screenings have certain limitations; while they can effectively identify most congenital deafness cases immediately after birth, they do not provide early warnings for late-onset or progressive hearing loss in children, nor for those with drug sensitivities. Deafness gene screening addresses these gaps and has proven beneficial. This study retrospectively analyzed the screening and diagnostic data of 10,754 newborns for hearing and deafness-related genes, yielding significant information for assessing the impact of genetic testing on the prevention and management of hereditary deafness, and offering a clinical foundation for selecting screening programs and strategies for preventing and treating hearing impairments.

## Materials and methods

The study involved newborns who underwent hearing and genetic screening for deafness at a Women’s Hospital between June 2020 and December 2021. Exclusion criteria included: ① Birth weight under 2500 g; ② Gestational age less than 37 weeks. A total of 10,754 newborns were included, comprising 5,648 males and 5,106 females. The study received approval from the Ethics Committee of the Women’s Hospital, and all procedures adhered to relevant guidelines and regulations. Ethics No.: (2016) Research Medical Ethics No. (001)-18. Prior to the genetic screening for hearing and deafness, informed consent was obtained from the guardians of all newborns.

Neonatal hearing screening was conducted following a strict protocol. The screening process consists of two stages: “primary screening and rescreening,” performed by healthcare professionals. TEOAE (MAICO Ero-SCAN, Diagnostics GmbH) and AABR (MAICO MB11, Diagnostics GmbH) devices were used. The primary hearing screening occurred 48–72 h after birth using transiently evoked otoacoustic emission (TEOAE) (MAICO Diagnostic GmbH Ero-SCAN type). If a newborn did not pass the monaural or binaural test, who were considered as failing. Newborns who did not pass the initial AABR screening (MAICO Diagnostic GmbH MB11) at 42 days post-delivery were also classified as failing. Those who failed the AABR were required to undergo more extensive hearing tests after three months, which included distortion product otoacoustic emission detection, acoustic impedance testing, auditory brainstem response detection, steady-state auditory evoked potential detection, and other assessments [5].

### Deafness genetic screening [6]

#### Sample preparation

Following the “Technical Specification for Screening of Newborn Diseases (2010 Edition),” peripheral blood was collected from the heel of the newborn using a special blood sampling card three days after birth to create three dried blood spots, each with a diameter of at least 8 mm. The newborn’s information was extracted from the delivery information system and recorded on the blood card, which was then packaged separately.

#### DNA extraction

A 3 mm diameter punch was taken from the dried blood spots, and DNA was extracted using a nucleic acid extraction kit (Chengdu Boao City Jingxing Biotechnology Co., LTD.). The concentration and purity of the DNA were assessed using a microspectrophotometer (model: Nanodrop2000, Thermo Fisher), with a minimum concentration requirement of 2–20 ng/μL.

#### PCR amplification

The prepared DNA (5 µL) was mixed with 20 µL of PCR amplification reagents. Three systems (A, B, C) were used for multiplex amplification of GJB2, GJB3, SLC26A4, and MT-RNR1 targets, as per the Jingxin 15 kit protocol. The Jingxin 15 kit utilizes three multiplex PCR systems (A, B, C) for targeted amplification of clinically significant variants in *GJB2*, *GJB3*, *SLC26A4*, and *MT-RNR1*. System A prioritizes high-frequency *GJB2* mutations (176del16, 235delC, 538C>T, 1226G>A) and mitochondrial *MT-RNR1* variants (1555A>G, splicing anomalies), with cross-validation via *SLC26A4* 1174A>T. System B focuses on *GJB2* splice-site defects (229-2A>G, 1229G>A) and mitochondrial *MT-RNR1* 1494C>T, overlapping with System A for shared loci (176del16, 538C>T) to ensure reproducibility. System C targets *GJB3* (35delG), *SLC26A4* splicing variants (IVS7-2A>G, IVS5+5G>A), and rare mitochondrial variants (2168A>G, 1755A>G). Cross-system validation through shared loci (e.g., IVS5+5G>A, 2027 M) enhances diagnostic reliability while minimizing interference. The mixture underwent multiplex PCR reaction in the gene amplification instrument ETC-811 Model: TC-96/G/H (b).

#### Detection method and result interpretation

The Jingxin 15 genetic deafness-related gene detection kit (using a microarray chip method) was employed to analyze 15 mutation sites associated with hereditary deafness. These include mutations such as 35delG, 176_191del16, 235del C, and 299_300del AT on the GJB2 gene, 538 C>T on GJB3, as well as several mutations on the SLC26A4 gene (2168 A>G, IVS7-2 A>G, 1174 A>T, 1226 G>A, 1229 C>T, IVS15+5 G>A, 1975 G>C, 2027 T>A) and mitochondrial MT-RNR1 (1494 C>T, 1555 A>G). The testing process involves using DNA as a template, amplifying and fluorescently labeling specific gene fragments with location-specific primers containing Tag sequences, hybridizing these with generic gene chips that have corresponding Tag sequences, and then scanning the results. The chip data analysis allows for the simultaneous detection of both wild-type (negative) and mutant (positive) results across the 15 loci, with confirmation of mutation sites through first-generation sequencing technology [7].

#### Statistical methods

Statistical analysis of the data was conducted using SPSS19.0 software. Count data were expressed as constituent ratios. The comparison of the deafness gene carrying rates among different hearing screening result groups was performed using the χ2 test, with a significance level set at p<0.05.

## Results

### Hearing screening results

Out of 10,754 newborns, 428 failed the initial hearing screening, resulting in a failing rate of 3.98 % (428/10,754). Among those, 259 were male infants (4.59 %, 259/5,648) and 169 were female infants (3.31 %, 169/5,106). Of the failing, 156 cases (36.45 %) involved both ears, 148 cases (34.58 %) involved the left ear, and 119 cases (27.80 %) involved the right ear. The 428 newborns who failed the initial screening underwent a second screening on day 42, with a passing rate of 85.51 % (366/428). Of those, 62 cases failed, including 27 cases (43.54 %) that failed both ears, 21 cases (33.87 %) that failed the left ear, and 14 cases (22.58 %) that failed the right ear.

### Deafness gene screening results

A total of 529 cases of deafness gene mutations were identified among the 10,754 newborns, resulting in an overall mutation carrying rate of 4.92 % (529/10,754). This included 307 cases (2.85 %, 307/10,754) of GJB2 gene mutations, 37 cases (0.34 %, 37/10,754) of GJB3 Double Heterozygous mutations, 162 cases (1.51 %, 162/10,754) of SLC26A4 gene mutations, and 35 cases (0.33 %, 35/10,754) of mitochondrial MT-RNR1 gene mutations. The specific mutation sites and types are detailed in Table 1.

**Table 1:** Type and number distribution of 15 deafness gene screening mutation loci in 10,754 neonates.

Gene	Genotype	Type	Case (n)	Frequency (%)
*GJB2*	35 del G	Heteroplasmy	1	0.19
	176_191 del 16	Heteroplasmy	17	3.21
	235 del C	Heteroplasmy	2	0.38
	235 del C	Heteroplasmy	242	45.75
	299_300 del AT	Heteroplasmy	35	6.62
*GJB3*		Heteroplasmy		
	538 C>T	Heteroplasmy	33	6.24
*SLC26A4*		Heteroplasmy		
	2168 A>G	Heteroplasmy	19	3.59
	IVS7-2 A>G	Heteroplasmy	110	20.79
	1174 A>T	Heteroplasmy	5	0.95
	1226 G>A	Heteroplasmy	7	1.32
	1229 C>T	Heteroplasmy	6	1.13
	IVS15+5 G>A	Heteroplasmy	2	0.38
	1975 G>C	Heteroplasmy	6	1.13
	2027 T>A	Heteroplasmy	2	0.38

*MT-RNR1*				

	1494 C>T	Homoplasmy	2	0.38
	1555 A>G	Homoplasmy	16	3.02
	1555 A>G	Homoplasmy	15	2.84

*GJB2/GJB2*				

	235delC/299delAT	Complex heteroplasmy	1	0.19

*GJB2/MT-RNR1*				

	235delC/1555A>G	Compound mutation	1	0.19
	176del16/1494 C>T	Compound mutation	1	0.19

*GJB2/SLC26A4*				

	235delc/IVS7 – 2A>G	Double heteroplasmy	2	0.38

*GJB2/GJB3*				

	299 del AT/538 C>T	Double heteroplasmy	1	0.19
	235delC/538 C>T	Double heteroplasmy	2	0.38
	176del16/538 C>T	Double heteroplasmy	1	0.19
Total			529	100.00

Results of hearing screening: 428 of the 10,754 newborns failed the initial hearing screening, and the failing rate of the initial screening was 3.98 % (428/10,754), of which 259 were male (4.59 %, 259/5,648) infants and females (3.31 %, 169/5,106). There were 156 cases (36.45 %) failed in both ears, 148 cases (34.58 %) failed in the left ear, and 119 cases failed in the suitable ear cases (accounting for 27.80 %). The 428 newborns who had failed the initial screening were screened a second time on day 42. The passing rate of the rescreening was 85.51 % (366/428), 62 cases failed, and 27 cases (43.54 %) failed both ears., The left ear failed in 21 cases (33.87 %), and the right ear failed in 14 cases (22.58 %). Deafness gene screening results in 529 cases of deafness gene mutations detected in 10,754 newborns. The overall deafness gene mutation carrying rate was 4.92 % (529/10,754), including 307 cases (2.85 %, 307/10,754) of *GJB2* gene mutation and *GJB3* 37 cases of heteroplasmy genetic mutation (0.34 %, 37/10,754), 162 cases of *SCL26A4* gene mutation (1.51 %, 162/10,754), 35 cases of mitochondrial *MT-RNR1* gene mutation (0.33 %, 35/10,754).

Findings from the combined hearing screening and deafness gene analysis revealed that 62 out of 10,754 newborns did not pass the hearing test. Among these 62 newborns, 7 were found to have mutations in deafness-related genes, representing 11.29 % (7/62). In contrast, of the 10,692 newborns who passed the hearing screening, 522 (4.88 %, 522/10,692) had mutations in the deafness gene. A statistically significant difference was observed in the mutation rates between the group that passed the hearing screening and the group that did not (χ2=5.412, p<0.05), as shown in Table 2.

**Table 2:** The results of newborn hearing concurrent genetic screening.

Hearing screening	Cases(n)	Genetic screening(n)		p_Value
		Referred	Passed	
Passed	10,692	522	10,170	0.032
Referred	62	7	55	

Results of joint screening of hearing screening and deafness genes, 62 of the 10,754 newborns failed hearing screening; of the 62 newborns who failed the hearing screening, 7 cases had deafness gene mutations, accounting for 11.29 % (7/62); among the 10,692 newborns who passed the hearing screening, 522 cases (4.88 %, 522/10,692) carried mutations in the deaf gene. There was a statistically significant difference in the carrying rate of deafness gene mutation between the hearing screening passed the group and the failing group (χ2=5.412, p<0.05).

## Discussion

Congenital deafness is among the most prevalent birth defects, alongside genetic predisposition. If not detected and addressed promptly, it can significantly hinder children’s language development, potentially resulting in deafness and muteness, which adversely impacts their quality of life. Statistics indicate that 1 in 1,000 of the 20 million newborns in China each year are born with congenital deafness [8]. When including cases of delayed and drug-induced deafness, approximately 60,000–80,000 new deaf individuals emerge annually, placing a substantial burden on families and society [9]. Currently, neonatal audiology screening has reduced the average age for diagnosing congenital deafness from 24 to 36 months to just 2–3 months [10], facilitating early clinical intervention and helping to preserve speech abilities and improve future quality of life. However, initial hearing screenings often yield a high rate of false positives [11]. For newborns who do not pass the screening, it is crucial to communicate the timing and nature of follow-up screenings to ensure timely diagnosis and treatment. This study revealed that the proportion of infants passing the screening was significantly higher than those passing the audiological assessment at 72 h after birth during a second evaluation at 42 days, indicating that the timing of the screening has a notable impact on results. Additionally, research showed that the passing rate for hearing screening was higher in the right ear compared to the left, and there was a greater prevalence of deafness among males than females, aligning with findings from other studies both domestically and internationally [12].

Newborn hearing screening is crucial for identifying immediate hearing impairments or potential deafness risks, but it is not effective in detecting late-onset, progressive, or drug-induced hearing loss at an early stage. Clinical practice and evidence-based medicine have shown that combining newborn hearing assessments with genetic testing for deafness offers significant advantages, particularly for late-onset deafness and mitochondrial gene mutations related to drug sensitivity. Genetic screening for deafness can also help identify reliable genetic causes in children with congenital hearing loss [13]. Research indicates that hereditary deafness in China is primarily caused by mutations in the GJB2, SLC26A4, GJB3, and 12 rRNA genes, and a comprehensive clinical pathway for deafness gene screening has been established [14]. In this study, deafness gene screening was performed using a microarray chip method, which is effective for large-scale screening due to the regional and ethnic variations in deafness gene mutations, focusing on 15 common mutation sites. The study identified 529 cases of deafness gene mutations among 10,754 newborns, resulting in a mutation carrier rate of 4.92 %. The most frequently observed mutations were GJB2 c.235delC (47.26 %) and SLC26A4 IVS7-2 A>G (21.17 %), aligning with previous research findings [15], 16]. Out of 62 newborns screened for common deafness genes, 55 had expected results. While genetic screening identifies at-risk infants, environmental factors and rare mutations necessitate ongoing audiological monitoring [17]. Thus, combined screening maximizes early detection of both congenital and late-onset cases. Without genetic testing, the causes of genetic deafness remain unclear, and mutation carriers may not receive necessary monitoring, potentially increasing the risk of deafness in future generations. Variants in the MT-RNR1 gene have been linked to aminoglycoside-induced hearing loss, showing variable penetrance and expressivity [18]. The study found a 6.61 % mutation rate in the MT-RNR1 gene among late-onset deafness cases, indicating that newborns carrying these mutations may be sensitive to specific aminoglycoside drugs, which serves as a warning for future hearing protection for the children and their families. There are also notable polymorphisms in the clinical presentation of hereditary deafness.

## Conclusions

Screening for genes associated with neonatal deafness can help identify the molecular causes of hearing loss and promptly detect newborns at high risk for hearing impairment. The increasing recognition of the detection rates of gene mutations linked to deafness in carriers, along with the clinical audiological features and interventions for homozygous or complex heterozygous mutations, has garnered clinical interest. Ongoing hearing screening and monitoring will provide long-term benefits for those who have undergone genetic screening for hearing loss. Implementing combined hearing and genetic screening for newborns can significantly enhance the efficiency of diagnosing neonatal deafness [19]. Most neonatal deafness gene mutations are inherited from parents, while a few result from new genetic mutations. For newborns with hearing loss due to a single hybrid gene locus mutation, diagnostic gene sequencing is essential to rule out rare mutations and accurately determine the cause of deafness, enabling tailored treatment. Research indicates that cochlear implants and hearing aids are the most effective solutions for hereditary deafness caused by gene mutations, improving the quality of life for affected individuals. Relying solely on hearing screening results may lead to overlooking many children at risk for delayed hearing impairment. Those who pass the hearing screening but carry deafness gene mutations, along with their future children, face a heightened risk of deafness and should be closely monitored. Integrating genetic and hearing screening enhances early diagnosis, though neither method alone suffices [20], 21]. Carriers of mutations require lifelong monitoring to mitigate environmental risks. In conclusion, genetic screening for deafness should be widely carried out in couples of childbearing age and neonates, so to achieve the goals of early detection, early intervention, early treatment, and effectively reduce the incidence of deafness and muteness [22].
